# Assessing basic and higher-level psychological needs satisfied through physical activity

**DOI:** 10.3389/fpsyg.2023.1023556

**Published:** 2023-02-20

**Authors:** Genevieve F. Dunton, Bridgette Do, Rachel Crosley-Lyons, Christine H. Naya, Micaela Hewus, Martina Kanning

**Affiliations:** ^1^Department of Population and Public Health Sciences, Keck School of Medicine, University of Southern California, Los Angeles, CA, United States; ^2^Department of Sport and Exercise Science, University of Stuttgart, Stuttgart, Germany

**Keywords:** psychological needs, physical activity, ecological momentary assessment, accelerometry, reliability, validity, scale development

## Abstract

**Background:**

There has been increasing interest in the extent to which the fulfillment of psychological needs is associated with physical activity engagement. However, a vast majority of studies consider only *basic* psychological needs such as relatedness, competence, and autonomy—with *higher-level* psychological needs such as challenge, creativity, and spirituality rarely being addressed. The aim of this study was to examine the preliminary reliability (i.e., internal consistency) and validity (i.e., discriminant, construct, and predictive) of a multi-dimensional scale to assess a range of basic and higher-level psychological needs satisfied through physical activity.

**Methods:**

A sample of 75 adults (ages 19–65 years, 59% female, 46% White) completed a baseline questionnaire measuring 13 psychological needs subscales (i.e., physical comfort, safety, social connection, esteem from others, individual esteem, learning, challenge, entertainment, novelty, creativity, mindfulness, aesthetic appreciation, and morality), exercise enjoyment, and exercise vitality. Participants then completed 14 days of accelerometer monitoring of physical activity and ecological momentary assessment of affective responses during physical activity sessions in daily life.

**Results:**

Internal consistency reliability was acceptable (>0.70) for all subscales except for mindfulness, aesthetic appreciation, and morality. Ten of the 13 subscales exhibited discriminant validity by differentiating between engagement (vs. no engagement) in at least one physical activity type (e.g., brisk walking and yoga/Pilates). All the subscales, except physical comfort and esteem from others, were associated with at least one of the construct validation criteria (e.g., exercise enjoyment, affective response during exercise). Five of the subscales were associated with at least one of the predictive validation criteria (i.e., light, moderate, vigorous intensity activity measured by accelerometer).

**Conclusion:**

Having the capacity to assess whether one’s current physical activity is failing to fulfill various psychological needs—combined with recommendations about which types of activities may satisfy those needs—may address an important gap in physical activity promotion.

## 1. Introduction

Although regular participation in physical activity leads to many health benefits (Hamasaki, 2016; Wahid et al., 2016; McTiernan et al., 2019), as little as 10% of U.S. adults engaged in recommended levels of ≥150 min/week of moderate-to-vigorous physical activity (Troiano et al., 2008). Progress in physical activity intervention research has stalled in recent years partially due to its focus on social and cognitive determinants of behavior—resulting in interventions that have modest effects on long-term physical activity change (Hartmann-Boyce et al., 2014; Gourlan et al., 2016). An area that has received increased attention in terms of potential influence on physical activity engagement is the extent to which behaviors are perceived as pleasurable (Rhodes and Kates, 2015). When individuals experience a pleasurable behavior, it is rewarding, and they are drawn to it in the future. However, an unpleasant experience can lead to future avoidance of that behavior (Dunton et al., 2020). Whether physical activity is perceived as pleasurable and rewarding may depend on the extent to which it fulfills the individual’s psychological needs (Teixeira et al., 2018). Indeed, the satisfaction of psychological needs is thought to lead to optimal well-being and positive emotional states (Deci and Ryan, 2002). Thus, satisfaction of psychological needs during physical activity offers a promising direction for physical activity intervention.

A variety of psychological needs have been studied in motivational psychology. Self-Determination Theory (SDT) suggests that intrinsic motivation for a behavior comes from satisfying basic psychological needs for relatedness, competence, and autonomy (Ryan and La Guardia, 2000). These needs from SDT fall in the middle of Maslow’s Hierarchy of Human Needs (Maslow, 1943). Maslow’s Hierarchy proposes a wider range of psychological needs, suggesting that humans behave to satisfy biological and safety needs at the lowest level, which are followed by needs for belongingness and esteem (similar to relatedness, competence, and autonomy from SDT), and then at the top of the hierarchy are higher-level needs for self-actualization (Deci and Ryan, 1985, 2000, 2008; Deci et al., 1991; Ryan and Deci, 2018). Although true self-actualization remains a somewhat elusive concept, it is thought to consist of “growth needs” that allow individuals to use and capitalize upon their unique preferences and abilities to reach their full potential (Goldstein, 1939; Whitehead, 2017). Higher-level psychological needs that have been proposed and studied in the self-actualization domain include creativity, challenge, learning, novelty, variety, entertainment and escapism, beauty and aesthetics, mindfulness and spirituality, and morality and altruism (Brock and Livingston, 2004; Gao et al., 2017; Sylvester et al., 2018; González-Cutre et al., 2020). Although lower-level and basic needs (e.g., biological, safety, belonging/relatedness, esteem/competence, and autonomy) are thought to be universally important for most people, the importance of these *higher-level* needs may vary depending on individual values and skills (Goldstein, 1939; Whitehead, 2017). Likewise, behaviors and experiences may differ in the extent to which they are able to satisfy higher-level psychological needs based on their purpose or context (Verstuyf et al., 2013; González-Cutre et al., 2020). Thus, providing tailored physical activity recommendations to fulfill higher-level psychological needs offers a critical missing link to understanding how to promote pleasurable experiences and sustained behavior engagement.

Although interest is growing in understanding how physical activity can fulfill psychological needs, a vast majority of studies consider only *basic* psychological needs derived from SDT (i.e., relatedness, competence, and autonomy; Wilson et al., 2003; Kirkland et al., 2011; Brunet et al., 2016; Kanning and Hansen, 2017; Teixeira et al., 2018; Antunes et al., 2020). This literature generally shows that satisfying needs for autonomy and competence through physical activity is associated with greater concurrent and prospective engagement (actual and self-reported) in physical activity behavior (Teixeira et al., 2012). Receiving much less attention, previous work has shown that physical activity can fulfill *higher-level* psychological needs for novelty (Fernández-Espínola et al., 2020; Engels et al., 2022), entertainment (Laor, 2020), creativity (Richard et al., 2018), and learning (Holt and Neely, 2011). Unlike basic psychological needs from SDT, the importance of these higher-level psychological needs may vary across people, and the extent to which these needs are satisfied may differ across types of physical activity (e.g., running, team sports, and hiking). However, to date, there are no existing validated measures that capture a full range of basic and higher-level psychological needs satisfied through physical activity. Having an instrument to capture which psychological needs are satisfied by what types of physical activity and for whom this occurs is an important step toward developing interventions in this area.

Thus, the overall objective of this study was to examine the preliminary reliability and validity of a multi-dimensional scale to assess a range of psychological needs satisfied during physical activity. The first aim was to identify a parsimonious set of internally-consistent of items to assess each of the proposed physical activity-related psychological need satisfaction dimensions. The outcome of this step was to narrow down the length of the target instrument (so that it could be reasonably administered without undue participant burden). The second aim was to evaluate discriminant validity by examining whether dimension-specific physical activity-related psychological need satisfaction differed across different types of physical activities. According to the conceptualization of self-actualization, higher-level psychological needs allow individuals to make the most of their unique preferences and abilities (Goldstein, 1939; Whitehead, 2017). Furthermore, activities may differ in the extent to which they are able to satisfy higher-level psychological needs depending on their purpose or context (Verstuyf et al., 2013; González-Cutre et al., 2020). Therefore, higher-level psychological needs (e.g., creativity, aesthetic appreciation, and morality), in particular, should differ in the extent to which they are satisfied across different types of activities. The third aim was to evaluate construct validity by examining whether dimensions of physical activity-related psychological need satisfaction were associated with exercise enjoyment, exercise vitality, and affective response during physical activity. The fourth aim was to assess predictive validity by examining whether dimensions of physical activity-related psychological need satisfaction were associated with device-based physical activity levels.

## 2. Materials and methods

### 2.1. Overview and design

This study used an observational design with a baseline questionnaire followed by 14 days of real-time monitoring and experience sampling during individuals’ daily lives using accelerometers and personal smartphones. No intervention was involved, and participants were asked to engage in their usual levels of physical activity. A 14-day protocol was used to capture a sufficient number of physical activity sessions during which affective responses could be measured while minimizing overall participant burden. Data were collected between August 2020 and 2021. Due to the federal, state, local, and university restrictions resulting from the COVID-19 pandemic, all study procedures were conducted in a fully remote manner.

### 2.2. Participants and recruitment

Potential participants were contacted through postings to social media and ResearchMatch (a national health volunteer registry that was created by several academic institutions and supported by the U.S. National Institutes of Health as part of the Clinical Translational Science Award program). Individuals previously participating in University of Southern California research studies were also contacted if they had indicated that they were interested in hearing about future research opportunities. Potential participants were asked eligibility screening questions online. Inclusion criteria were as follows: (1) 18–65 years old; (2) currently engage in ≥60 min of structured physical activity per week; (3) able to speak and read English; and (4) use an Android smartphone as the primary personal phone. Exclusion criteria were: (1) cardiovascular, respiratory, muscular, or bone/joint problems that preclude physical activity; (2) inability to answer smartphone-based surveys for extended periods of time due to work, caregiving, or driving requirements; (3) a body mass index <18 or > 37.5 kg/m^2^ (categorized as underweight or morbidly obese); (4) been treated for cancer within the past 6 months; (5) current cigarette smoker; (6) diabetic; (7) unable or unwilling to answer smartphone-based surveys while exercising; (8) take psychotropic medications; or (9) receive treatment for any psychiatric disorder. Additionally, participants who swam for physical activity more than once a week were excluded because the accelerometer device could not be worn in the water. If participants were eligible, a videoconference appointment was made to obtain informed consent.

### 2.3. Procedures

All procedures were conducted in accordance with the Declaration of Helsinki and approved by the University of Southern California institutional review board for the study of human subjects. During the initial videoconference session, participants were consented for the study and provided their mailing address. Following this session, participants received an electronic link to complete the online baseline questionnaire (on their own time) assessing satisfaction of psychological needs through physical activity, types of physical activity, physical activity level, exercise enjoyment and vitality, demographics, and other psychological factors. After successful completion of the baseline questionnaire, a waist-worn accelerometer was mailed to the participant and a videoconference orientation session was scheduled. During the orientation session, study staff provided instructions on how to download the study smartphone application and complete the study’s real-time monitoring procedures of responding to smartphone-based Ecological Momentary Assessment (EMA) surveys (Shiffman et al., 2008) and wearing an accelerometer over the next 14 days. Individuals were compensated up to $150 for participating in the study with incentives based upon compliance to study procedures.

### 2.4. Measures

#### 2.4.1. Satisfaction of psychological needs through physical activity

The investigators developed an original instrument to assess the satisfaction of basic and higher-level psychological needs. A top-down approach was taken in which 13 different psychological need dimensions were identified *a priori*, which mapped onto Maslow’s Hierarchy of Human Needs and SDT (i.e., physical comfort, safety, social connection, esteem from others, individual esteem, learning, challenge, entertainment, novelty, creativity, mindfulness, aesthetic appreciation, and morality). For each dimension, the investigators then generated a range of potential items. The item generation process was intended to be creative and avoid redundancy by developing items that conveyed unique concepts. This step was intended to be as open-ended and unconstrained as possible to foster the creative process. No limits were imposed on the maximum number of items to be generated. The larger list of items was then narrowed down based on face validity (i.e., most closely represented the intended psychological need dimension). Face validity is a relatively quick and straightforward way of determining whether items seem to be useful when first looking at them. This step involved removing items that did not appear to be relevant or appropriate on the surface (e.g., incorrect use of synonyms or poor fitting examples) for measuring the intended construct. In addition, redundant items were further excluded, and grammatical corrections were made. Lastly, items that referred to specific types and amounts of physical activity were removed to make the instrument applicable to a wide range of populations. After these steps, a total of 65 face-valid items remained with each psychological need dimension being assessed by 2–13 items (e.g., feeling discomfort, doing something dangerous, cooperating with others, showing off skills, mastering tasks, doing things I have not done before, using my imagination, focusing on the present moment, learning something new, being challenged, being entertained, being in nature, and helping people; see Supplementary material).

Participants in the current study completed this 65-item instrument to measure satisfaction of psychological needs during physical activity. They were asked to “Think about any moderate or vigorous physical activity or exercise (including brisk/fast walking, classes, and sports) that you have done over the past 4 weeks.” Using a nine-point response scale ranging from 1 = not at all to 9 = a lot, participants evaluated the extent to which their recent physical activity involved the satisfaction of basic and higher-level psychological needs in the 13 dimensions.

#### 2.4.2. Types of physical activity

Participants reported usual types of physical activity performed. Survey instructions asked, “For the physical activity that you just described (in the last 7 days), please indicate what type of exercises you did/typically do (select all).” Response options included: brisk/fast walking, jogging or running, yoga or Pilates, hiking, and team sports (e.g., soccer, basketball, and football). Other types of physical activity (including dance, elliptical machine, swimming, bicycling or cycling, weight-lifting or strength training, and other) were also assessed but not used in the current analyses due to low frequency of reporting.

#### 2.4.3. Exercise enjoyment

Enjoyment was assessed with the Physical Activity Enjoyment Scale (PACES; Kendzierski and DeCarlo, 1991). The questionnaire provided the instructions, “Please rate how you feel about the moderate or vigorous physical activity or exercise (including brisk/fast walking, classes, and sports) you have been doing in the past 4 weeks.” This statement is followed by 18 items that are answered on a seven-point bipolar response scale (e.g., 1 = I enjoy it to 7 = I hate it, 1 = I feel bored to 7 = I feel interested, and 1 = I dislike it to 7 = I like it). Eleven items are reverse-scored. Higher scores indicate greater enjoyment. In the present study, PACES had high internal consistency (Cronbach’s α = 0.81).

#### 2.4.4. Exercise vitality

Vitality describes a state of feeling alive, energized and alert that is thought to be a component of eudaimonic well-being (Ryan and Deci, 2001). As such, vitality contributes to psychological well-being. Vitality through exercise was assessed using a four-item version of the Subjective Vitality Scale (Ryan and Frederick, 1997). Participants were given the statement, “When I engaged in physical activity or exercise (including brisk/fast walking, classes, and sports) in the past 4 weeks…,” which was followed by the responses: I felt alive and vital; I felt so alive I just wanted to burst; I had energy and spirit; and I felt energized. Each response was scored on a seven-point Likert scale ranging from 1 = not at all to 7 = very true. In the present study, this scale had high internal consistency (Cronbach’s α = 0.85).

#### 2.4.5. Affective response during physical activity *via* EMA

Participants completed EMA surveys through a commercial smartphone application that had been downloaded to their personal smartphone at the beginning of the study (movisensXS by movisens GmbH; Karlsruhe, Germany). Participants were asked to engage in their usual levels of physical activity during the 14-day study. Immediately before each naturally-occurring physical activity bout, participants were instructed to manually press a button in the EMA app to indicate the start of a physical activity session. The EMA app then automatically prompted a *during-physical activity* survey 15 min later. The first item asked, “Are you finished exercising?” If the response was “no,” items assessing momentary affect were triggered and the app automatically promoted another during-physical activity survey 15 min later (30 min after the start of the physical activity session). If the participant indicated that they were still not finished exercising at that point, another during-physical activity survey was prompted after 45 min (75 min after the start of the physical activity session). Answering “yes” to having finished physical activity led to a separate post-physical activity EMA survey not included in the present analyses. Each *during-physical activity* survey assessed affective response by asking, “How are you feeling right now?” with items measured through a digital visual analog semantic differential sliding scale, which were converted to scores from 0 to 100 based on distance between the anchor points. Four affective response items were used, which mapped onto core affective valence (Bad-Good), energetic arousal (Exhausted-Energized), activated negative and positive affect (Miserable-Thrilled), and interest (Bored-Interested; Russell, 1980; Thayer, 1990; Wilhelm and Schoebi, 2007; Wolff et al., 2021). An item measuring tense arousal (Relaxed-Nervous) was also assessed but not included in the current analyses given the lack of hypothesized relation with psychological needs satisfaction through physical activity. Each EMA survey required about 30 s to complete.

#### 2.4.6. Device-based physical activity level

Physical activity level was also measured using a waist-worn Actigraph, Inc., GT3X model accelerometer at a frequency of 30 Hz with a 30-s epoch. The device was worn on the right hip, attached to an adjustable belt, at all times except while sleeping, bathing, or swimming. Periods of non-wear (>60 continuous minutes of zero activity counts) and non-valid days (<10 h of wear) were not included in the analyses. A custom-built R program applied cut points for time spent in light, moderate, and vigorous intensity physical activity based on national data (Troiano et al., 2008; Belcher et al., 2010) and were generated from the Freedson prediction equation (Freedson et al., 1998, 2005; Roemmich et al., 2000; Harrell et al., 2005; Belcher et al., 2010; Laska et al., 2012). Time spent in light, moderate, and vigorous physical activity was summed for total physical activity and divided by the number of minutes of valid device wear time to calculate the percent if valid wear time spent engaging in physical activity.

#### 2.4.7. Participant characteristics

Participants self-reported the following characteristics: age, gender identity, race, ethnicity, total family income for the past 12 months, marital status, educational attainment, number of children, and employment status. Participants were not required to answer these questions if they preferred not to.

### 2.5. Statistical analyses

Prior to analyses, all inversely worded items were reverse-coded. Variables were screened for normality. As a result, vigorous intensity physical activity measured by accelerometer was log transformed to address positive skew. Light and moderate intensity physical activity met normality assumptions and were not transformed.

The first aim was to identify a parsimonious set of items to assess each of the *a priori* identified 13 psychological needs dimensions. To do so, we eliminated items from each subscale that did not favorably contribute to scale reliability measured by internal consistency (i.e., how closely related a set of items are as a group). Using SPSS (Version 28.0.0.0), internal consistency reliability was generated by entering all items (ranging from 2 to 13 items per subscale) hypothesized to assess a needs satisfaction dimension. Cronbach’s alpha coefficients, which are a function of the number of items and the average inter-correlations among items, were calculated for each subscale. Cronbach’s alpha “if item deleted” values were used to monitor improvements in internal consistency reliability associated with one-at-a-time stepwise removal of each item from the subscale. The item whose removal contributed to the greatest improvement in Cronbach’s alpha was eliminated at each step. When stepwise removal no longer improved the Cronbach’s alpha, the procedure was halted. Means were calculated for the remaining set of items representing each subscale, which were used in further analyses. Additional descriptive statistics, including standard deviations, were generated for the final set of items included in each subscale. Bivariate intercorrelations among the subscales were also estimated.

The second aim was to evaluate discriminant validity by examining whether subscales of psychological needs satisfied through physical activity differed across types of physical activities that individuals perform. Independent samples *t*-tests compared the means of each needs satisfaction subscale between individuals who did versus did not report engaging in various types of physical activity (i.e., brisk/fast walking, jogging or running, yoga or Pilates, hiking, and team sports) over the past 7 days. Separate *t*-tests were run for each activity type. As an additional conservative measure, Bonferroni corrections for family-wise error rates were applied (13 tests per activity type, α = 0.05/13 = 0.004).

The third aim was to evaluate construct validity by examining whether psychological needs satisfied through physical activity were associated with exercise enjoyment, exercise vitality, and affective response during physical activity. Bivariate correlations tested the extent to which each psychological needs subscale was associated with exercise enjoyment and vitality. The associations between each psychological needs subscale and affective responses during exercise (good-bad, energized-exhausted, thrilled-miserable, and interested-bored) were also examined.

The fourth aim was to assess predictive validity by examining whether psychological needs satisfied through physical activity are associated with device-based physical activity levels. Bivariate correlations tested whether each psychological needs subscale was related to levels of physical activity over the next 14 days.

## 3. Results

### 3.1. Data availability

A total of 75 participants were enrolled in the study. Participants were missing data on the psychological needs dimensions as follows: mindfulness (*n* = 1), individual esteem (*n* = 2), and physical comfort (*n* = 2). Furthermore, two participants were missing data on the exercise enjoyment scale, and one participant was missing data on the exercise vitality scale. A total of 66 individuals responded to EMA prompts, of which 59 individuals provided data for at least one EMA prompt during physical activity (*n* = 584 prompts total across all participants; *M* = 9.92, SD = 8.0, range = 1–40 prompts per participant). There were 47 participants who responded to at least one EMA prompt +30 min into the physical activity bout (*n* = 214 prompts total across participants; *M* = 4.55, SD = 3.18, range = 1–14 prompts per person). All 75 participants had complete data on the types of physical activity performed in the past 7 days. A total of 64 participants had at least one valid day (≥10 h) of accelerometer data (*M* = 11.47, range = 1–14 days per person). With casewise deletion of missing data, the sample sizes ranged from 45 to 75 across the analyses.

### 3.2. Demographics

Of the total sample (*N* = 75), 8% were Hispanic White, 18% African-American, 21% Asian, 46% were Non-Hispanic White, and 7% more than one race. Ages ranged from 19 to 65 years (*M* = 39.8, SD = 13.0 years). Participants identified as 58.7% female, 38.7% male, and 2.6% trans/non-binary. In terms of their marital status, 34% were married, 11% were divorced or separated, 15% were members of an unmarried couple, 39% were never married, and 1% widowed. The highest level of educational attainment was as follows: 4% high school, 13% some college, 83% college degree or higher. Among the total sample, 68% were employed for wages or self-employed, 8% were unemployed, 16% were students, 4% were homemakers, and 4% retired. Sixty-five percent of participants had one or more children. The annual household income breakdown was as follows: 25% < $35,000, 31% ≥ $35,000 and < $65,000, 21% ≥ $65,000 and < $105,000, and 23% ≥ $105,000.

### 3.3. Descriptive statistics for validation criteria

Of the total sample (*N* = 75), 67% reported engaging in brisk walking for physical activity in the past 7 days, 39% engaged in jogging or running, 28% did yoga or Pilates, 23% went hiking, and 7% performed team sports. The average scores for exercise enjoyment and exercise vitality were 5.09 (SD = 0.76) and 5.61 (SD = 0.91), respectively, on a seven-point scale. The average scores for the affective response during exercise scales from EMA were as follows (*n* = 47): Good-Bad (*M* = 78.68, SD = 13.34), Energized-Exhausted (*M* = 71.18, SD = 16.50), Thrilled-Miserable (*M* = 73.55, SD = 13.08), and Interested-Bored (73.83, SD = 16.24) on a 0–100 scale. Of the 64 participants for whom accelerometer data were available, engagement in physical activity was as follows: light (*M* = 179.0 min/day, SD = 46.14), moderate (*M* = 33.66 min/day, SD = 22.67), and vigorous (median = 1.33 min/day, SD = 8.89).

### 3.4. Descriptive statistics and internal consistency of the psychological needs satisfaction subscales

Table 1 shows descriptive statistics for the psychological needs satisfied through physical activity subscales after removal of items not contributing favorably to internal consistency. Means, standard deviations, and Cronbach’s alphas for the remaining items in each subscale are shown. The stepwise item removal process left each subscale with 2–3 items (33 items total; see Table 2). A reliability coefficient of 0.70 or higher is considered “acceptable” in most social science research (Taber, 2018). Cronbach’s alphas were greater than 0.70 for all subscales except mindfulness, aesthetic appreciation, and morality. Subscales that were most highly endorsed (i.e., highest mean values) were physical comfort and safety needs satisfied through physical activity, and the least highly endorsed were receiving esteem from others and social connection through physical activity.

**Table 1:** Descriptive statistics for psychological needs satisfied through physical activity.

Sub-scale	Num. items	*M* (SD)	Cronbach’s alpha	*N*
Physical comfort	2	6.76 (1.84)	0.825	73
Safety	2	8.65 (0.79)	0.850	75
Social connection	3	3.67 (2.38)	0.876	75
Esteem from others	3	3.28 (2.03)	0.797	75
Individual esteem	3	5.42 (2.08)	0.813	73
Learning	2	5.23 (2.23)	0.773	75
Challenge	3	4.76 (2.02)	0.769	75
Entertainment	2	5.33 (2.07)	0.742	75
Novelty	3	4.57 (1.99)	0.818	75
Creativity	3	4.96 (2.35)	0.863	75
Mindfulness	3	5.09 (2.00)	0.673	74
Aesthetic appreciation	2	5.07 (2.02)	0.516	75
Morality	2	4.71 (2.52)	0.696	75

Results generated after stepwise deletion of items not contributing to increasing Cronbach’s alphas. Response scales ranges from 1 = not at all to 9 = a lot.

**Table 2:** Satisfaction of psychological needs through physical activity instrument (33 items).

Sub-scale	Items
Physical comfort	Feeling physical discomfort (R)
	Feeling exhaustion (R)
Safety	Feeling unsafe (R)
	Feeling threatened (R)
Social connection	Being a part of a team
	Cooperating with others
	Being with other people
Esteem from others	Receiving praise from others
	Being recognized for what I’ve done
	Showing off my skills
Individual esteem	Being competent at something
	Mastering challenging tasks
	Accomplishing difficult things
Learning	Building my skills
	Learning something new
Challenge	Being challenged
	Taking risks
	Solving problems
Entertainment	Being interested
	Being entertained
Novelty	Doing things I have not done before
	Being curious about things
	Exploring things
Creativity	Using my imagination
	Being playful
	Expressing my emotions
Mindfulness	Connecting to my spirituality
	Relaxing
	Focusing on the present moment
Aesthetic Appreciation	Being in nature
	Going places that I have not gone before
Morality	Helping other people
	Doing the right thing

Instructions: “Think about any moderate or vigorous physical activity or exercise (including brisk/fast walking, classes, sports) that you have done over the past 4 weeks. Please rate the extent to which those physical activities involved the following things. 1 = Not at all….0.9 = A lot.” Results generated after stepwise deletion of items not contributing to increasing Cronbach’s alphas. R= reverse coded.

### 3.5. Intercorrelations among psychological needs satisfied through physical activity

Table 3 shows intercorrelations among the psychological needs satisfied through physical activity subscales. The satisfaction of psychological needs for physical comfort and safety through physical activity subscales only had small correlations with the other subscales. Satisfying needs for social connection and esteem from others through physical activity were moderately positively associated with the other subscales except mindfulness and aesthetic appreciation. Satisfying needs for individual esteem, learning, challenge, novelty, creativity, and entertainment were either higher or moderately positively correlated each other. Satisfying psychological needs for mindfulness, aesthetic appreciation, and mindfulness were moderately positively correlated with each other and most other subscales.

**Table 3:** Intercorrelations among psychological needs satisfied through physical activity.

Sub-scale	Physical comfort	Safety	Social connection	Esteem from Others	Individual esteem	Learning	Challenge	Entertainment	Novelty	Creativity	Mindfulness	Aesthetic appreciation	Morality
Physical Comfort	**--**												
Safety	**0.228**	**--**											
Social Connection	**−0.168**	**−0.284**	**---**										
Esteem from Others	**−0.163**	**−0.290**	**0.653**	**---**									
Individual Esteem	**−0.214**	**−0.062**	**0.446**	**0.505**	**---**								
Learning	**−0.085**	**−0.087**	**0.562**	**0.601**	**0.787**	**---**							
Challenge	**−0.201**	**−0.160**	**0.676**	**0.660**	**0.801**	**0.779**	**---**						
Entertainment	**−0.078**	**−0.073**	**0.591**	**0.559**	**0.706**	**0.656**	**0.745**	**---**					
Novelty	**−0.007**	**−0.065**	**0.577**	**0.446**	**0.542**	**0.612**	**0.718**	**0.719**	**---**				
Creativity	**−0.023**	**−0.133**	**0.670**	**0.638**	**0.666**	**0.717**	**0.813**	**0.752**	**0.771**	**---**			
Mindfulness	**0.020**	**0.089**	**0.350**	**0.316**	**0.571**	**0.545**	**0.529**	**0.666**	**0.516**	**0.664**	**---**		
Aesthetic appreciation	**−0.027**	**−0.113**	**0.331**	**0.442**	**0.270**	**0.337**	**0.460**	**0.515**	**0.551**	**0.484**	**0.464**	**---**	
Morality	**−0.103**	**−0.202**	**0.627**	**0.639**	**0.514**	**0.487**	**0.611**	**0.573**	**0.457**	**0.636**	**0.511**	**0.414**	**---**

**Light gray** = small correlation (*r*’s = 0.01–0.39), **gray** = moderate correlation (*r*’s = 0.40–0.69), and **bolded** = large correlation (*r*’s = 0.70–0.99). ns = 71–75.

### 3.6. Discriminant validity

**Figure 1:**
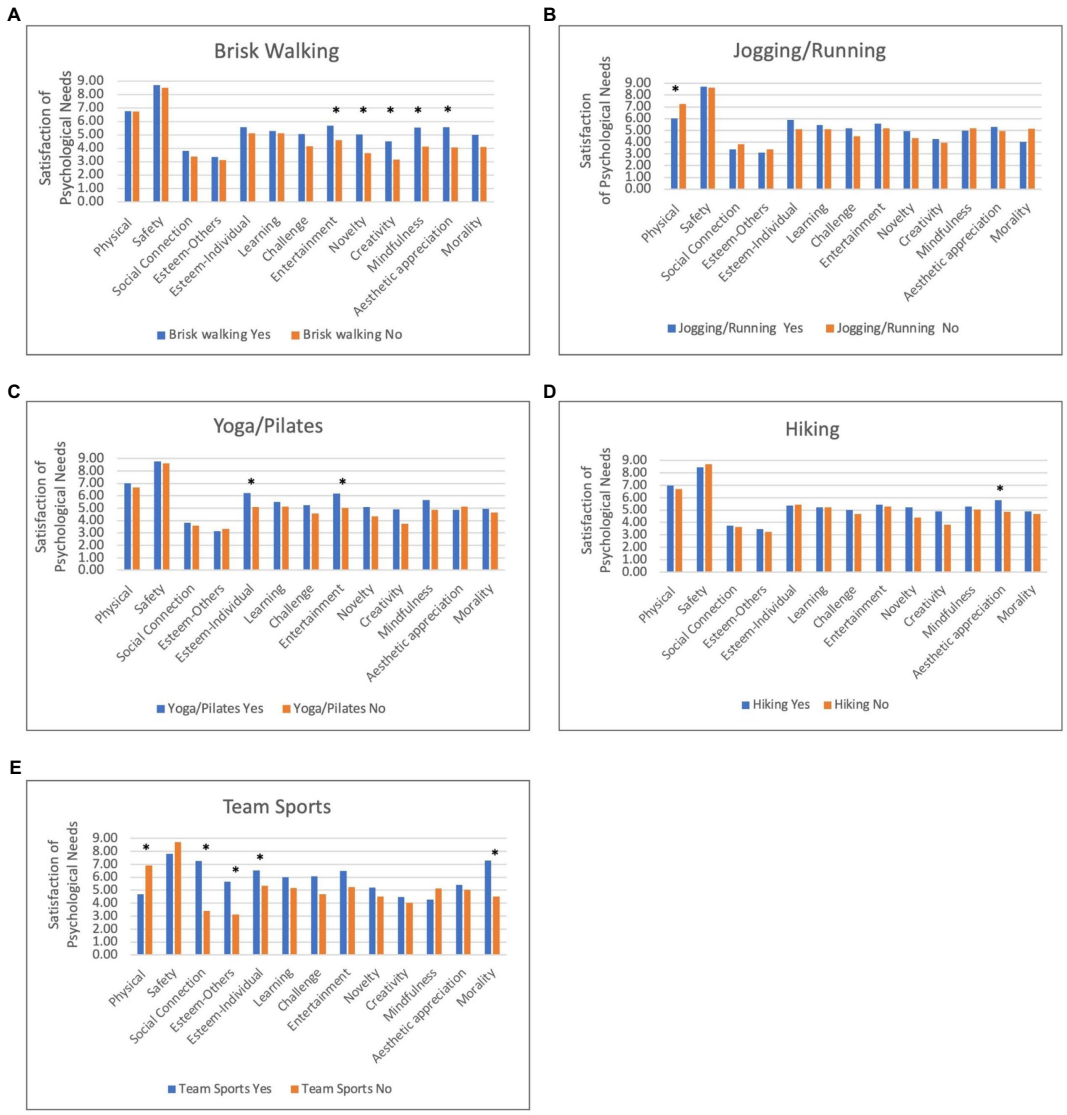
Differences in related psychological need satisfaction by past 7-day engagement (yes vs. no) in different types of physical activity: **(A)** Brisk Walking; **(B)** Jogging/Running; **(C)** Yoga/Pilates; **(D)** Hiking; and **(E)** Team Sports. ^*^p < 0.05.

To test discriminant validity, we examined whether satisfaction of different psychological needs varied across types of physical activity. Figure 1 shows differences in the scores for the satisfaction of psychological needs subscales by participation (yes vs. no) in a range of physical activity types. Individuals who engaged in brisk walking reported greater satisfaction of needs for entertainment, novelty, creativity, mindfulness, and aesthetic appreciation than individuals who did not engage in brisk walking. Participation in jogging or running was associated with less satisfaction of physical comfort needs. Furthermore, individuals who participated in yoga or Pilates had greater satisfaction of needs for individual esteem and entertainment than those who did not participate in yoga or Pilates. Hiking was associated with greater satisfaction of needs for aesthetic appreciation. Lastly, participants who engaged in team sports reported lower satisfaction of physical comfort needs, and higher needs satisfaction for social connection, individual esteem, esteem from others, and morality through physical activity. After Bonferroni corrections were applied, differences in novelty, mindfulness, and aesthetic appreciation for brisk walking and differences in social connection and morality for team sports remained significant.

### 3.7. Construct validity

To test construct validity, we examined associations of psychological needs satisfaction through physical activity with questionnaire-reported exercise enjoyment and vitality, and various affective responses measured through EMA during physical activity. Table 4 shows that satisfying psychological needs for safety, individual esteem, entertainment, and mindfulness through physical activity were positively associated with exercise enjoyment. Satisfying psychological needs for social connection, individual esteem, learning, challenge, entertainment, novelty, creativity, mindfulness, aesthetic appreciation, and morality through physical activity were positively related to exercise vitality. Table 5 shows that satisfying psychological needs for entertainment and mindfulness were positively associated with feeling good during physical activity. Furthermore, satisfying psychological needs for social connection, learning, entertainment, novelty, and creativity were associated with feeling more energized (than fatigued), thrilled (than miserable), and interested (than bored) during physical activity. Additionally, satisfying psychological needs for individual esteem was associated with feeling more energized (than fatigued) and interested (than bored) during physical activity. Also, satisfying psychological needs for challenge was associated with feeling more energized (than fatigued) and thrilled (than miserable) during physical activity. Lastly, mindfulness was associated with feeling more interested (than bored) during physical activity.

**Table 4:** Bivariate correlations of psychological needs satisfied through physical activity with exercise enjoyment and exercise vitality.

Sub-scale	Exercise enjoyment		Exercise vitality	
	*r*	*p*	*r*	*p*
Physical comfort	0.205	0.087	0.010	0.932
Safety	0.263	**0.025**	0.085	0.470
Social connection	0.084	0.479	0.254	0**.029**
Esteem from others	0.016	0.890	0.193	0.100
Individual esteem	0.239	**0.045**	0.296	**0.001**
Learning	0.207	0.079	0.335	**0.004**
Challenge	0.116	0.330	0.316	**0.006**
Entertainment	0.295	**0.011**	0.352	**0.002**
Novelty	0.138	0.243	0.325	**0.005**
Creativity	0.196	0.097	0.377	**0.001**
Mindfulness	0.334	**0.004**	0.462	**<0.001**
Aesthetic appreciation	0.117	0.325	0.231	**0.048**
Morality	0.129	0.278	0.246	**0.035**

Values of *p* < 0.05 are bolded. ns = 72–74.

**Table 5:** Bivariate correlations of psychological needs satisfied through physical activity with affective responses during physical activity measured through ecological momentary assessment.

Sub-scale	Good-Bad		Energized-Exhausted		Thrilled-Miserable		Interested-Bored	
	*r*	*p*	*r*	*p*	*r*	*p*	*r*	*p*
Physical comfort	0.127	0.074	0.119	0.431	0.181	0.228	0.111	0.464
Safety	0.120	0.423	0.155	0.297	0.118	0.430	0.142	0.342
Social connection	0.263	0.074	0.300	0.**041**	0.367	0.**011**	0.308	0.**035**
Esteem from others	0.002	0.991	0.086	0.567	0.056	0.056	0.072	0.630
Individual esteem	0.235	0.121	0.378	**0.009**	0.217	0.153	0.294	**0.050**
Learning	0.273	0.063	0.363	**0.012**	0.335	**0.021**	0.408	**0.004**
Challenge	0.186	0.211	0.333	**0.022**	0.293	**0.046**	0.270	0.067
Entertainment	0.385	**0.008**	0.434	**0.002**	0.390	**0.007**	0.398	**0.006**
Novelty	0.187	0.208	0.373	**0.010**	0.354	**0.015**	0.328	**0.025**
Creativity	0.257	0.082	0.350	**0.016**	0.373	**0.010**	0.355	**0.014**
Mindfulness	0.402	**0.006**	0.274	0.065	0.276	0.063	0.323	**0.028**
Aesthetic appreciation	−0.127	0.395	−0.062	0.681	−0.124	0.408	−0.142	0.339
Morality	0.085	0.568	0.107	0.475	0.100	0.503	0.087	0.559

Values of *p* < 0.05 are bolded. ns = 45–47.

### 3.8. Predictive validity

Predictive validity was assessed by examining whether psychological needs satisfied through physical activity were associated with reported and monitor-based physical activity levels. Table 6 shows that satisfying psychological needs for entertainment, novelty, and mindfulness were positively associated with light intensity physical activity. Satisfying psychological needs for aesthetic appreciation through physical activity were positively associated with moderate intensity physical activity. Lastly, satisfying psychological needs for physical comfort through physical activity was negatively associated with vigorous intensity physical activity. Satisfying psychological needs for safety, social connection, esteem from others, individual esteem, learning, entertainment, creativity, and morality were not significantly associated with any of the physical activity outcome measures.

**Table 6:** Bivariate correlations of psychological needs satisfied through physical activity with device-based physical activity behavior.

Sub-scale	Light intensity/valid minute		Moderate intensity/valid minute		Vigorous intensity/valid minute	
	*r*	*p*	*r*	*p*	*r*	*p*
Physical comfort	0.046	0.723	0.021	0.869	−0.300	**0.018**
Safety	0.024	0.851	0.033	**0.**798	0.107	0.401
Social connection	0.130	0.305	0.032	0.802	0.008	0.952
Esteem from others	0.075	0.555	0.103	0.419	0.135	0.288
Individual esteem	0.169	0.184	−0.073	0.572	0.069	0.590
Learning	0.225	0.074	−0.044	0.731	0.104	0.414
Challenge	0.188	0.137	0.060	0.637	0.102	0.424
Entertainment	0.330	**0.008**	−0.100	0.994	0.120	0.347
Novelty	0.247	**0.049**	0.135	0.287	0.160	0.205
Creativity	0.168	0.184	0.029	0.822	0.014	0.913
Mindfulness	0.285	**0.024**	0.022	0.800	0.073	0.571
Aesthetic appreciation	0.186	0.141	0.249	**0.047**	0.189	0.135
Morality	0.105	0.407	−0.011	0.933	−0.067	0.597

Physical activity was assessed over 7 days. Valid minute indicates the time that the accelerometer device was worn. Vigorous intensity has been log transformed to adjust for non-normality. *p* values < 0.05 are bolded. ns = 63–64.

## 4. Discussion

The aim of this study was to examine the preliminary reliability and validity of a multi-dimensional scale to assess a range of psychological needs satisfied through physical activity. This instrument improves upon existing measures of psychological needs satisfaction through physical activity by assessing a combination of both basic (e.g., physical comfort, safety, social connection, esteem from others, and individual esteem) and higher-level (e.g., learning, challenge, entertainment, novelty, creativity, mindfulness, aesthetic appreciation, and morality) needs. Understanding how physical activity may fulfill a range of different psychological needs could facilitate greater understanding of how to promote rewarding experiences and sustained physical activity behavior engagement.

Overall, analyses yielded a parsimonious set of reliable items for each of the 13 subscales measuring the satisfaction of psychological needs through physical activity. After stepwise removal of items not favorably contributing to internal consistency, most psychological needs subscales were comprised of 2–3 items with acceptable reliability (Cronbach’s alpha >0.70; Taber, 2018). The exceptions were the mindfulness, aesthetic appreciation, and morality subscales, whose reliabilities were moderate (range 0.516–0.696). Some caution may need to be exerted when interpreting findings from these three subscales—especially the aesthetic appreciation scale, which had the lowest reliability—or authors may otherwise choose not to use them. Following what would be expected from Maslow’s Hierarchy of Human Needs, participants in this sample rated that physical activity most highly satisfied their needs for physical comfort and safety. Maslow’s Hierarchy suggests that these two basic needs must be satisfied before other higher-level needs can be pursued (Deci and Ryan, 1985, 2000, 2008; Deci et al., 1991; Ryan and Deci, 2018). Surprisingly, the least satisfied psychological needs subscales in the current sample were receiving esteem from others and social connection through physical activity. Both Maslow’s Hierarchy and SDT would suggest that these types of social needs, similar to belongingness and relatedness, are quite important to achieving well-being and successful behavioral engagement (Deci and Ryan, 1985, 2000, 2008; Deci et al., 1991; Ryan and La Guardia, 2000; Ryan and Deci, 2018). However, the current study occurred during the year and a half of the COVID-19 pandemic (Aug 2020–2021). Therefore, COVID recommendations for social-distancing were in effect during much of the study, which may have limited social interactions during physical activity. Also, outside of COVID, individuals may generally find it difficult to find or arrange for others (including friends and family members) to accompany them during physical activity sessions due to conflicting interests, varying abilities, or scheduling problems (Kerr and Hertel, 2011). The lower levels of satisfaction of social needs through physical activity may be indicative of a problem that should be addressed on a population level to promote healthy levels of physical activity engagement. Physical activity interventions and programs may benefit from specifically seeking out ways for participants to satisfy needs for social interactions and connection to others.

Findings supported the discriminant validity of the subscales by showing that psychological needs satisfied through physical activity vary across different types of physical activities. For example, the fact that brisk walking satisfied needs for entertainment, novelty, creativity, mindfulness, and aesthetic appreciation whereas jogging and running failed to satisfy any psychological needs suggests that unique aspects of these forms of physical activity (such as intensity, muscle groups involved, and context) may fulfill different psychological wants and desires. Lower intensity activities such as brisk walking may provide opportunities for mindfulness and creativity because they require less physical exertion and attention (Yogev et al., 2008). Further evidence for discriminant validity of the subscales was shown by the association of yoga and Pilates with the fulfillment of individual esteem and entertainment needs. The strengthening, flexibility, and balance skills utilized by the types of body movements in yoga and Pilates may satisfy individual esteem needs because they allow opportunities to feel competent and master tasks (Lopes et al., 2019). Given that hiking is a type of activity that necessitates being outdoors, the satisfaction of psychological needs for appreciation through it was consistent with expectations. Discriminant validity of the subscales was particularly salient with regard to participation in team sports, as it was the only type of activity that satisfied needs for social connection, esteem from others, and morality. Interestingly, participating in running and jogging, and team sports was associated with lower satisfaction of physical comfort needs, suggesting that those types of activities may be riskier or more physically demanding. Overall, the pattern of discriminant associations indicates not all psychological needs are equally satisfied across types of activities.

To examine the construct validity, we examined whether psychological needs satisfied through physical activity were associated with exercise enjoyment, exercise vitality, and affective response during physical activity. The extent to which satisfaction of psychological needs through physical activity leads to more pleasant experiences while exercising supports the overarching theoretical framework driving this research question (Ryan and Deci, 2001; Deci and Ryan, 2002). Almost all the subscales were positively associated with exercise vitality whereas a smaller subset (i.e., safety, individual esteem, entertainment, and mindfulness) were associated with exercise enjoyment. This pattern of findings suggests that the satisfaction of psychological needs through physical activity may be more closely linked to pleasant physical experiences such as feeling alive, energized, and alert, as measured by the exercise vitality scale. In contrast, more cognitive- and attention-focused psychological needs subscales (e.g., entertainment and mindfulness) were associated with aspects of the physical activity experience that involved evaluations and judgments (e.g., like it, feel interested) measured by the exercise enjoyment scale. Interestingly, entertainment and mindfulness were the only psychological needs subscales that were associated with the affective response during physical activity item measuring core affective valence (i.e., Good-Bad). However, most of the satisfaction of psychological needs subscales were positively associated with the affective response during physical activity items that reflected affective arousal (i.e., Energized-Exhausted), activated positive and negative affect (i.e., Thrilled-Miserable), and interest (i.e., Interested-Bored). The overall pattern of these findings suggests that the fulfillment of psychological needs does contribute to pleasant experiences during physical activity, especially when those experiences are assessed in real-time during real-world physical activity situations as opposed to retrospective evaluative judgments of past physical activity.

The final goal of this paper was to evaluate predictive validity by examining whether psychological needs satisfied through physical activity were associated with device-based physical activity levels. The purpose of this analysis was to examine whether the fulfillment of psychological needs leads to greater engagement in the target behavior, as theory and prior evidence would suggest (Wilson et al., 2003; Gholidahaneh et al., 2020). Satisfying needs for physical comfort was negatively associated with levels vigorous intensity physical activity. These findings suggest that exercising in a way that meets needs for physical comfort (e.g., avoiding pain and exhaustion) most likely involves shorter and lower intensity physical activity sessions. Furthermore, satisfying needs for entertainment, novelty, and mindfulness may be positively associated with light intensity physical activities because they allow for greater attention to the present surroundings (Yogev et al., 2008) and opportunities to explore new environments and types of movement (Hull and Stewart, 1995; Lakicevic et al., 2020). Similar to the discriminant validity results described above, aesthetic appreciation was associated with higher self-reported levels of moderate intensity activity, most likely because walking can more easily increase from light to moderate intensity while outdoors. Overall, linkages between the various psychological needs subscales (i.e., novelty, mindfulness, and aesthetic appreciation) and device-measured physical activity further support their validity in predicting the target behavior.

### 4.1. Strengths and limitations

Strengths of this study included the use of real-time and device-based measures of validation criteria, as well as a relatively diverse study sample in terms of age, race, and income levels. In contrast, limitations included missing data and a relatively modest sample size, which did not permit confirmatory factor analyses and could primarily detect moderate to large effect sizes. However, the goal of this study was to shorten the length of the target instrument (so that it could be reasonably administered without undue participant burden) and provide preliminary evidence of its reliability and validity—both of which can be accomplished with relatively modest sample sizes. Another potential limitation is the lower reliability of the mindfulness, aesthetic appreciation, and morality subscales, which may influence the statistical conclusion validity of the later analyses using those subscales. In the current paper, lower reliability of those subscales may bias later correlation coefficients toward the null hypothesis. Further work using larger and more heterogeneous samples is needed to test its factor structure and test–retest reliability, and to provide more evidence of validation using additional measures of physical activity experiences and behavior. In particular, whether the instrument is reliable and valid in child and adolescent populations needs to be studied.

## 5. Conclusion

This study provided preliminary evidence of the reliability and validity of a multi-dimensional scale to assess a range of basic and higher-level psychological needs satisfied through physical activity. This instrument addresses a critical gap in the research literature that is a lack of available measures of higher-level psychological needs. Having the capacity to assess whether one’s current physical activity is failing to fulfill basic and higher-level psychological needs may have important applications in clinical and intervention settings. For example, the tool can be used by therapists or fitness professionals to determine whether one’s current physical activities are satisfying their psychological needs. If not, it may explain why an individual is having difficulty achieving physical activity goals or why various intervention strategies are not working. A novel adaptive physical activity promotion strategy would be to recommend alternative types of activities to better satisfy those needs and test again at a later time. This type of information can serve as the basis for intervention programs and physical activity prescriptions that are individually-tailored to meet psychological needs with the goal of promoting more pleasant experiences and sustainable behavior.

## Data availability statement

The raw data supporting the conclusions of this article will be made available by the authors, without undue reservation.

## Ethics statement

The studies involving human participants were reviewed and approved by University of Southern California Institutional Review Board. The patients/participants provided their written informed consent to participate in this study.

## Author contributions

GD wrote the first draft of the manuscript and performed the statistical analysis. GD, BD, and MK contributed to the conception and design of the study. BD, RC-L, CN, MH, and MK contributed to manuscript editing and revision, and GD incorporated final edits. All authors contributed to the article and approved the submitted version.

## Funding

Funding for this research was provided by the University of Southern California Zumberge Large Interdisciplinary Grant.

## Conflict of interest

The authors declare that the research was conducted in the absence of any commercial or financial relationships that could be construed as a potential conflict of interest.

## Publisher’s note

All claims expressed in this article are solely those of the authors and do not necessarily represent those of their affiliated organizations, or those of the publisher, the editors and the reviewers. Any product that may be evaluated in this article, or claim that may be made by its manufacturer, is not guaranteed or endorsed by the publisher.
