# Supplementary material for: Assessing basic and higher-level psychological needs satisfied through physical activity
Source: Front Psychol. 2023 Feb 20;14:1023556. doi: 10.3389/fpsyg.2023.1023556 (PMC9986481; doi:10.3389/fpsyg.2023.1023556)
Supplement: Supplementary file 1 [file Table_1.DOCX]

Supplementary Material

# Supplementary Figures and Tables

## Supplementary Tables

**Supplementary Table 1.**

Satisfaction of Psychological Needs Through Physical Activity Items (65 items)

*Think about any moderate or vigorous physical activity or exercise (including brisk/fast walking, classes, sports) that you’ve done over the past 4 weeks. Please rate the extent to which those physical activities involved the following things.*

|  | 1 Not at all  2  3 A little  4  5 Moderately  6  7 Quite a bit  8  9 A lot |
| --- | --- |
| 1. Expressing myself artistically |  |
| 1. Receiving praise from others |  |
| 1. Feeling unsafe |  |
| 1. Winning at things |  |
| 1. Feeling physical pain |  |
| 1. Being in nature |  |
| 1. Playing games |  |
| 1. Doing things I haven’t done before |  |
| 1. Being curious about things |  |
| 1. Feeling supported by others |  |
| 1. Feeling physically sick |  |
| 1. Distracting myself |  |
| 1. Being alone |  |
| 1. Connecting to my spirituality |  |
| 1. Being successful |  |
| 1. Doing meaningful things |  |
| 1. Being competent at something |  |
| 1. Going places that I haven’t gone before |  |
| 1. Socializing |  |
| 1. Exerting physical effort |  |
| 1. Getting rewards |  |
| 1. Using my imagination |  |
| 1. Looking good |  |
| 1. Being challenged |  |
| 1. Being recognized for what I’ve done |  |
| 1. Building my skills |  |
| 1. Being embarrassed |  |
| 1. Mastering challenging tasks |  |
| 1. Accomplishing my goals |  |
| 1. Being a part of a team |  |
| 1. Doing things for no apparent reason |  |
| 1. Being incompetent at something |  |
| 1. Doing things independently |  |
| 1. Being creative |  |
| 1. Relaxing |  |
| 1. Learning something new |  |
| 1. Being bored |  |
| 1. Losing at things |  |
| 1. Feeling exhaustion |  |
| 1. Competing against others |  |
| 1. Accomplishing difficult things |  |
| 1. Being in love |  |
| 1. Receiving criticism from others |  |
| 1. Playing or listening to music |  |
| 1. Showing off my skills |  |
| 1. Being rejected by others |  |
| 1. Failing to accomplish my goals |  |
| 1. Cooperating with others |  |
| 1. Being playful |  |
| 1. Taking risks |  |
| 1. Being interested |  |
| 1. Exploring things |  |
| 1. Expressing my emotions |  |
| 1. Feeling safe |  |
| 1. Feeling physical discomfort |  |
| 1. Focusing on the present moment |  |
| 1. Being with other people |  |
| 1. Being unsuccessful |  |
| 1. Feeling physical pleasure |  |
| 1. Feeling threatened |  |
| 1. Being entertained |  |
| 1. Solving problems |  |
| 1. Helping other people |  |
| 1. Doing the right thing |  |
| 1. Doing something dangerous |  |

*Scoring*

*Physical Needs: 5, 11, 20, 39, 59*

*Safety/Security Needs: 3,54, 60, 50, 65*

*Relatedness Needs: 10, 13, 19, 30, 42, 46, 48, 57*

*Esteem- Social Needs: 2, 23, 25, 27, 40, 43, 45*

*Esteem-Individual Needs: 4, 7, 15, 17, 21, 28, 29, 32, 33, 38, 41, 47, 58*

*Novelty- 8, 9, 18, 52,*

*Creativity- 1, 22, 34, 44, 48, 53*

*Mindfulness/Spirituality: 12, 14, 35, 56*

*Learning: 26, 36*

*Challenge: 24, 50, 62*

*Entertainment: 37, 51, 61*

*Morality: 63, 64*
